# Congenital toxoplasmosis in the United States: clinical and serologic findings in infants born to mothers treated during pregnancy

**DOI:** 10.1051/parasite/2019013

**Published:** 2019-03-06

**Authors:** Tudor Rares Olariu, Cindy Press, Jeanne Talucod, Kjerstie Olson, José Gilberto Montoya

**Affiliations:** 1 Palo Alto Medical Foundation Toxoplasma Serology Laboratory Palo Alto CA USA; 2 Department of Medicine and Division of Infectious Diseases and Geographic Medicine, Stanford University School of Medicine Stanford CA USA; 3 Victor Babes University of Medicine and Pharmacy Timisoara Romania

**Keywords:** Congenital toxoplasmosis, Infants, Laboratory diagnosis, Serologic findings, Clinical findings

## Abstract

We assessed clinical and serologic findings in 25 infants with congenital toxoplasmosis born to mothers treated during pregnancy in the United States. Results indicate a lower prevalence of eye findings and hydrocephalus in the group of infants born to treated mothers (62.5% and 38.5%, respectively) compared to results on the same pathologies reported in our previous cohort of infants born to untreated mothers (92.2% and 67.7%, respectively). The sensitivity of the IgM ISAGA and IgA ELISA in the present study were lower (44% and 60%, respectively) compared to sensitivity of these methods in our previously studied group of infants born to untreated mothers (86.6% and 76.5%, respectively). These findings provide further evidence that anti-parasitic treatment if administered during pregnancy can contribute to better clinical outcomes, even in countries where systematic screening and treatment have not been routinely implemented.

## Abstract (fr)

Nous avons évalué les résultats cliniques et sérologiques chez 25 nourrissons atteints de toxoplasmose congénitale, nés de mères traitées pendant la grossesse aux États-Unis. Les résultats indiquent une prévalence plus faible des lésions oculaires et de l’hydrocéphalie dans le groupe des nourrissons nés de mères traitées (62,5 % et 38,5 %, respectivement) par rapport aux résultats sur les même pathologies rapportés dans notre cohorte précédente de nourrissons nés de mères non traitées (respectivement 92,2 % et 67,7 %). La sensibilité des tests ISAGA IgM et ELISA IgA dans la présente étude était inférieure (44 % et 60 %, respectivement) par rapport à la sensibilité de ces méthodes dans notre groupe de nourrissons nés de mères non traitées (respectivement 86,6 % et 76,5 %). Ces résultats confirment également que le traitement antiparasitaire administré pendant la grossesse peut contribuer à améliorer les résultats cliniques, même dans les pays où le dépistage et le traitement systématiques n’ont pas été mis en œuvre de manière systématique.

## Introduction

Recent studies have further demonstrated that screening and treatment for toxoplasmosis during pregnancy result in a decrease of mother to child transmission and clinical sequelae [1, 3, 5, 6, 13, 17]. Effective anti-*Toxoplasma* treatment, if administered early during pregnancy, has been shown to significantly decrease vertical transmission as well as to improve clinical outcomes [17]. Researchers have estimated that prenatal treatment can reduce the risk of serious neurological sequelae of congenital toxoplasmosis by 75% [3].

We have previously shown that congenitally infected infants born to untreated pregnant women in the United States may present major clinical signs including severe toxoplasmosis [10]. We therefore undertook this study to assess clinical and serologic findings in congenitally infected infants, born to treated mothers during their pregnancy in the United States, during the same time period of our untreated cohort.

## Study population and methods

The Palo Alto Medical Foundation Toxoplasma Serology Laboratory (PAMF-TSL) database was retrospectively searched for data on infants (from birth to 180 days of age) in whom congenital toxoplasmosis was suspected or confirmed, who had been tested for *Toxoplasma gondii*-specific IgG, IgM and IgA antibodies between January 1991 and December 2005, and whose mothers received anti-*Toxoplasma* treatment. This period was selected in order to match that of a previous study reported by our group in infants born to untreated mothers. The criteria for diagnosis of congenital toxoplasmosis were as previously reported [10]: Infants who were IgG antibody positive in the Sabin-Feldman dye test (DT) and who met at least 1 of the following criteria: (1) presence of IgM and/or IgA *T. gondii* antibodies after day 10 of life (if serum was obtained before 10 days of life, a follow-up confirmatory serum sample was obtained); (2) persistence of IgG antibodies in the DT by 12 months of age; (3) presence of IgM antibodies in cerebrospinal fluid (CSF); and (4) positive polymerase chain reaction (PCR) results or isolation of *T. gondii* from amniotic fluid (AF), CSF or blood, or positive PCR results in urine.

Twenty-five congenitally infected infants were identified as born to mothers who received anti-*Toxoplasma* treatment during pregnancy. In these 25 infants, sera were submitted because the prenatal diagnosis of acute acquired infection had been suspected in the mother by the primary care provider and had been confirmed at PAMF-TSL. Anti-*Toxoplasma* therapy was administered to these acutely infected pregnant women, as previously described [14]. However, detailed information regarding gestational age of maternal infection, timing and doses of the anti-*Toxoplasma* drugs used during gestation (*e.g.* spiramycin, pyrimethamine/sulfadiazine/folinic acid, or spiramycin followed by pyrimethamine/sulfadiazine/folinic acid) was not available to PAMF-TSL.

We sought to determine whether clinical and serologic findings at birth were different between the cohorts of infants born to treated *vs.* untreated mothers, despite the limited information in the history of anti-*Toxoplasma* treatment. The untreated cohort comprised 164 infants born to mothers untreated during pregnancy [10]. In the untreated cohort, infants also did not receive post-natal treatment before the diagnosis of congenital toxoplasmosis was confirmed [10] (Table 1). In the treated cohort (current study), post-natal treatment was initiated in all infants at birth and the majority of these infants were tested shortly after birth (Table 1). The previously reported untreated cohort [10] and the treated cohort were referred to PAMF-TSL through the same mechanisms and referral network. Thus, both cohorts were subject to the same biases and limitations. Of note, in both the current report and previously published report [10], we could not assess the long-term sequelae and long-term serologic test results in infants with congenital toxoplasmosis. For the purpose of these studies, we did not perform serologic or clinical follow-up of the infected infants. The main reason for this is that we were consultants for the laboratory confirmation of the diagnosis and were not actively involved in the management of these patients. Unfortunately, we were not asked to perform routine serial serologic or clinical follow-up of the infected infants. Patients with several serum samples tested in our laboratory were sent to us when the samples were needed for laboratory confirmation of congenital toxoplasmosis. Therefore, clinical manifestations and serologic results of infants included in both cohorts represent the findings reported at the time the first serum sample was collected for testing at PAMF-TSL.

**Table 1:** Characteristics of infants with congenital toxoplasmosis born to treated and untreated mothers in the United States.

Characteristics	Treated	Untreated
Mothers tested for *T. gondii* during pregnancy	Yes	No
Diagnosis of acute toxoplasmosis during pregnancy	Yes	No
Mothers treated for *T. gondii* during pregnancy	Yes	No
Serologic testing for *T. gondii* IgG, IgM, IgA antibodies performed after birth	Yes	Yes
Clinical signs reported after birth	Yes	Yes
Missing information on clinical signs and serologic test results in some infants	Yes	Yes
Infants in the United States referred to PAMF-TSL for laboratory confirmation of congenital toxoplasmosis	Yes	Yes
Post-natal treatment before diagnosis of congenital toxoplasmosis was confirmed	Yes, at birth	No

Similar to the previous report on the untreated cohort [10], when the data were analyzed in infants with major clinical findings, such as eye disease, calcifications and hydrocephalus, the number of infants in the denominator was always corrected to account for the number of infants in whom clinical and imaging examinations were performed and information was available. Similarly, when data were analyzed for serologic tests, the number of infants in the denominator was always corrected to account for the number of infants in whom the test was performed [10].

The age given for each infant is the age when the first serum sample was drawn for testing at PAMF-TSL. Serologic test results are solely those obtained in the first serum submitted for testing to PAMF-TSL. All serologic and PCR tests were performed at PAMF-TSL, as previously reported [10]. Serologic tests included the Sabin-Feldman dye test (DT) for *T. gondii* IgG antibodies, IgM ISAGA, IgA ELISA, and IgE ELISA. The following titers were considered positive: IgM ISAGA, 3–12; IgA ELISA ≥ 1.0; and IgE ELISA ≥ 1.9 [10].

The 25 congenitally infected infants were born to 23 mothers. Two pairs were twins. Serologic tests in mothers included the DT, IgM ELISA, IgA ELISA, the differential agglutination (AC/HS) test, and the VIDAS IgG avidity test (Biomérieux, Marcy l’Étoile, France) [8].

PCR was performed using two target sequences from the 35-fold repetitive B1 gene [4, 11]. In 2002, the PCR method was modified to use real-time technology on the Applied Biosystems Sequence Detection System (ABI PRISM 7700 and 7000; Carlsbad, CA), and was performed according to the manufacturer’s recommendations. When samples were available, PCR was performed in AF, CSF, whole blood, or urine. The PAMF Institutional Review Board approved our study of stored samples and associated clinical and laboratory information.

### Statistical analysis

Data were analyzed using the EPI INFO statistical package (version 3.3.2; CDC, Atlanta, GA). Mantel-Haenszel chi-square and the 2-tailed Fisher test were used to compare proportions between groups. A probability level of *p* < 0.05 was considered to indicate statistical significance.

## Results

### Clinical findings

The 25 infants ranged in age from 0 to 127 days (average 21 days), 9 (36%) were males. Clinical information was available for 19 (76%) of the 25 infants. In 16 (84%) of the 19 infants, one or more severe clinical manifestations of congenital toxoplasmosis were noted. For infants in whom clinical investigations were performed and information was available, eye disease was described in 10 (62.5%) of 16 infants, cerebral calcifications in 13 (76.5%) of 17 infants, and hydrocephalus in 5 (38.5%) of 13. In 3 (27.3%) of the 11 infants in whom information was available for each of these manifestations, eye findings, cerebral calcifications and hydrocephalus were reported to be present concurrently (Table 2).

**Table 2:** Serologic and clinical findings in infants with congenital toxoplasmosis born to treated and untreated mothers in the United States.

		No. of infants aged 0–180 days*		*p* value	(95% CI)
		Treated (*n* = 25)	Untreated (*n* = 164)		
Serologic findings	IgM(+)/IgM performed (%)	11/25 (44)	142/164 (86.6)	<0.001	(0.04–0.3)
	IgA(+)/IgA performed (%)	15/25 (60)	127/164 (77.4)	0.06	(0.18–1.05)
	IgE(+)/IgE performed (%)	3/16 (18.7)	41/102 (40.2)	0.1	(0.09–1.28)
	Eye disease/*n* (%)	10/16 (62.5)	119/129 (92.2)	0.003	(0.04–0.46)
Clinical findings	Cerebral calcifications/*n* (%)	13/17 (76.5)	94/118 (79.7)	0.75	(0.24–2.77)
	Hydrocephalus/*n* (%)	5/13 (38.5)	67/99 (67.7)	0.039	(0.09–0.98)
	E + C + H/*n* (%)	3/11 (27.3)	53/86 (61.6)	0.048	(0.05–0.94)

* Age when first serum was drawn for testing. E: eye disease, C: calcifications, H: hydrocephalus, *n*: number of evaluated infants, CI: confidence interval for odds-based parameters.

### Serologic findings

IgM antibodies were demonstrated in 11 (44%) of the 25 infants (Table 1). IgA antibodies were demonstrated in 15 (60%) of the 25 infants, and in 6 (24%) they were noted in the absence of demonstrable IgM antibodies. Positive IgA test results were detected only in neonates and their rate (75%) was higher compared to the positive IgM test results (50%) in the same age group. Both IgM and IgA antibodies were detected only in neonates, 9 (45%) of 20. Neither IgM nor IgA antibodies were demonstrated in 8 (32%) of the 25 infants.

### Comparison of clinical and serologic findings in infants born to treated and untreated mothers

There was a significantly lower frequency of eye disease and hydrocephalus in infants who were born to treated mothers versus those born to untreated mothers (*p* = 0.003 and *p* = 0.039, respectively) (Table 2). In addition, the presence of these three major clinical findings concurrently was less frequently reported in infants of treated mothers compared to those of untreated mothers (*p* = 0.048).

Comparative evaluation of serologic test results indicates that IgM antibodies were also present less frequently in infants of treated (44%) than of untreated mothers (86.6%) (*p* < 0.001). No significant differences between infants born to treated and untreated mothers were noted when we evaluated the presence of *T. gondii* IgA or IgE antibodies (*p* = 0.06 and *p* = 0.1). Both IgM and IgA antibodies were significantly less commonly detected in infants born to treated mothers (36%) than in those born to untreated mothers (70.7%) (*p* < 0.001) (Table 3).

**Table 3:** IgM ISAGA and IgA ELISA *Toxoplasma* antibody test results in infants with congenital toxoplasmosis in the United States.

Infants (No. tested)	Number of infected infants with detectable *T. gondii* antibodies (%)			
	IgM*	IgA	IgM or IgA*	IgM(+) IgA(+)*
Born to treated mothers (25)	11 (44)	15 (60)	17 (68)	9 (36)
Born to untreated mothers (164)	142 (86.6)	127 (77.4)	153 (93.3)	116 (70.7)

* Differences are statistically significant at *p* < 0.05.

When we analyzed the prevalence of demonstrable IgA antibody in the absence of IgM antibody, we found that this was significantly higher in infants born to treated mothers (24%) versus those born to untreated mothers (6.7%) (*p* = 0.01) (data not shown). The number of infants in whom IgM and IgA antibodies were not detectable was significantly higher in the infants born to treated mothers (32%) compared to those born to untreated mothers (6.7%) (*p* < 0.001) (data not shown).

## Discussion

Findings in the present study suggest a lower prevalence of eye findings (62.5%) and hydrocephalus (38.5%) in the group of infants born to treated mothers compared to that reported in our previous cohort of infants born to untreated mothers: 92.2% had eye disease and 67.7% had hydrocephalus. A significant difference was also observed when the presence of eye findings, calcifications and hydrocephalus was reported concurrently, with a 27.3% frequency in the group of infants born to treated mothers versus 61.6% in the untreated cohort.

The clinical outcomes in the present study can be compared with those formerly observed in Europe after treatment of infected mothers was instituted during pregnancy. In France, for instance, the frequency of severe toxoplasmosis diagnosed in newborns decreased remarkably after 1978, when systematic serologic screening and treatment for *T. gondii* infection acquired in pregnancy was introduced [12, 16]. Since then, many European countries have reported lower frequencies of eye findings and intracranial calcifications following implementation of systematic prenatal screening programs and prophylaxis in pregnant women identified as having acquired *T. gondii* infection during pregnancy [15].

Moreover, hydrocephalus and other systemic signs such as splenomegaly or pneumonia have not been reported from Western European studies in recent years. Importantly, the European studies were prospective and the mothers were systematically screened and treated during pregnancy [12].

The low frequency of clinical findings observed in these recent European cohorts contrasts with the higher frequency of symptoms in children observed in earlier reports at times before systematic serologic screening of pregnant women and treatment during pregnancy were implemented [2].

One of the limitations of our study was that the gestational age at which maternal infection was acquired was unknown; gestational age at time of maternal infection is known to have a marked impact on the rate of clinical findings in infected infants [12]. It has been shown that the earlier infection occurs during pregnancy, the higher the likelihood of clinical signs; in contrast, the opposite is true later in pregnancy, and infected infants have a more favorable prognosis [17]. Thus, although unlikely, we cannot rule out entirely the possibility that the treated cohort of infants could have a higher proportion of mothers who were infected later in pregnancy.

In addition, information regarding timing and doses of the anti-*Toxoplasma* drugs used during pregnancy were not available. Similar to the previous report on the untreated cohort [10], we have some infants in whom information on the clinical signs and serologic tests results were not available to us. However, both cohorts went through an identical referral process to PAMF-TSL [10]. Thus, data from this study suggest that treatment administered during pregnancy may contribute to a better clinical outcome in infants, since the prevalence of eye disease and hydrocephalus in these groups of patients was significantly reduced.

The sensitivities of the IgM ISAGA and IgA ELISA in the present study were lower (44% and 60%, respectively) compared to the IgM ISAGA and IgA ELISA sensitivity in our previously studied group of infants born to untreated mothers (86.6% and 76.5%, respectively). Similar trends of lower frequency of IgM and IgA detection in treated infants, compared to those untreated, have been reported in Europe (25% and 85% for IgM, and 57% and 80% for IgA, respectively) [7]. These findings suggest that prenatal treatment decreases the sensitivity of serologic tests for diagnosis of congenital toxoplasmosis in the neonatal period.

Our study suggests that anti-parasitic treatment if administered during pregnancy contributes to a better clinical outcome in infected infants at the price of diminished sensitivity in serological tools for diagnosis. Similar results have previously been reported in countries where screening programs and treatment protocols were implemented [3, 5, 6, 13, 15, 17]. In these countries, anti-*Toxoplasma* treatment, if instituted early *in utero* during the gestational period, has been shown to significantly improve clinical outcomes in the infected infants at the expense of lower frequency in the detection of neonatal IgM and IgA [7]. Therefore, screening programs for *T. gondii* in pregnant women and therapy for women who acquire toxoplasmosis during pregnancy should be implemented in the United States to decrease vertical transmission, the frequency of clinical signs, and the occurrence of severe congenital toxoplasmosis [9].

## Conflict of interest

The authors declare that they have no conflicts of interest in relation to this article.
